# Effect of athletic fatigue damage and the associated bone targeted remodeling in the rat ulna

**DOI:** 10.1186/s12938-017-0384-1

**Published:** 2017-08-08

**Authors:** Li Hao, Li Rui-Xin, Han Biao, Zhao Bin, Hao Bao-Hui, Liu Ying-Jie, Zhang Xi-Zheng

**Affiliations:** 1 Institute of Medical Equipment, Academy of Military Medical Sciences, Tianjin, China; 2 Department of Orthopaedics Trauma, First Hospital of Jilin University, Changchun, China

**Keywords:** Athletic injuries, Fatigue, Bone remodeling, Microcracks, Mechanical property

## Abstract

**Background:**

Fatigue damage of the long bones is prevalent in running athletes and military recruits due to vigorous mid- and long-term physical activity. The current study attempted to know the features of bony athletic fatigue damage and to explore the mechanism of fatigue damage repair through bone targeted remodeling process.

**Methods:**

Right ulnae of the Wistar rats were fatigue loaded on an INSTRON 5865 to construct the athletic fatigue damage model, and several time points (i.e. experimental days: 0, 7, 13 and 19) were selected to simulate physiological status, preliminary, mid-term and perennial stage during continuous high-intensive training, respectively. The multi-level responses of rat ulnae under the athletic fatigue loading, including cellular protein expression, micro damage or micro-crack and macro mechanical properties, were tested and statistically analyzed.

**Results:**

Wistar rats, subjected to the athletic fatigue loading protocol, experienced a decrease of ulna fatigue mechanical properties and an active bone resorption of the loaded ulnae in the early stage, whereafter, a hyperactive bone formation and significant improvements of ulnae fatigue mechanical properties were detected. However, a deterioration of quasi-static mechanical properties in the subsequent period implied limitations of bone remodeling to maintain the bearing capacity of bone during long-term strenuous exercise.

**Conclusions:**

In summary, after athletic fatigue loading, bone targeted remodeling is activated and proceeds to repair fatigue damage, but only to a certain extent.

## Background

Bones experience various mechanical environments in daily human activities, with exceptional mechano-sensitivity resulting from bone remodeling by an activity “balance” between osteoblasts (OBs) and osteoclasts (OCs) [1–3]. A large proportion (approximately 70%) of bone remodeling is stochastic, participating in biochemical regulation and balancing calcium salt, and the other 30% is targeted remodeling, which is specialized to repair damage or microcracks in bones according to their load situation and demands [4–6]. There are common or universal mechanisms of bone damage and targeted remodeling processes among species that are based on the local strain or stress distribution [1, 7].

Long bone fatigue damage is prevalent in running athletes and military recruits due to vigorous mid- and long-term physical activity [8, 9]. It is characterized as generation, accumulation and coalescence of microcracks; the deterioration of mechanical properties; or even stress fractures [10, 11]. Loading intensity, frequency (f), and number of cycles (N) are critical factors that influence bone fatigue damage [12, 13], and there also seems to be a threshold of local peak strain that leads to destructive bone damage and bearing invalidation [14–16].

In the present study, we hypothesized that 8000 cycles of dynamic loading producing a 3000 με peak strain on the rat ulna could imitate athletic fatigue damage and activate the bone targeted remodeling process, and based on our earlier experiments, we constructed an athletic fatigue damage model of the rat ulna, and attempted to explore the features of bony athletic fatigue damage and the mechanism of fatigue damage repair through bone targeted remodeling process.

## Methods

### Animals and materials

Female Wistar rats (3 months old) were obtained from the laboratory animal center of the Academy of Military Medical Sciences in Beijing, China. The experiments performed were within the animal welfare regulations and guidelines for the Academy of Military Medical Sciences. ELISA Kits for rat estradiol (E2), bone gamma-carboxyglutamic-acid-containing protein (BGP) and tartrate-resistant acid phosphatase 5b (TRAP-5b) were purchased from Cloud-Clone Corporation in Wuhan, China.

### Experimental design

**Fig. 1:**
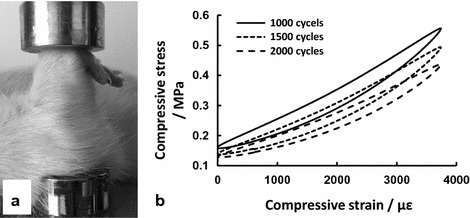
The athletic fatigue loading of rat ulna (**a**) and compressive σ–ε curves recording (**b**) on an INSTRON 5865

A total of 33 female Wistar rats were randomly divided into four groups by athletic fatigue loading duration, including a physiological intensity group (i.e. Group Day 0 or the Control group, 9 rats) and three high-intensity groups (Group Day 7, Day 13 and Day 19; 8 rats/group). Rats in the control group were fed normally with no fatigue loading, while the right ulnae of the rats in the high-intensity groups were fatigue-loaded on an INSTRON 5865 with general anesthesia every other day for 8000 cycles at a frequency of 1.5 Hz and a constant strain amplitude of 3000 με over a period of 20 days (a total of nine loading days starting from the second day), and data of σ–ε were recorded every 500 loading cycles (Fig. 1). Samples of rat ulnae and serum were obtained from the rats sacrificed on day 7 (Group Day 7), day 13 (Group Day 13) and day 19 (Group Day 19). Histological and morphometric analyses were performed using hematoxylin–eosin (HE) staining on non-decalcified ulna slices. Concentrations of serum proteins (E2, BGP and TRAP-5b) and mechanical properties were also analyzed.

### Development of the athletic fatigue damage model in the rat ulna

**Fig. 2:**
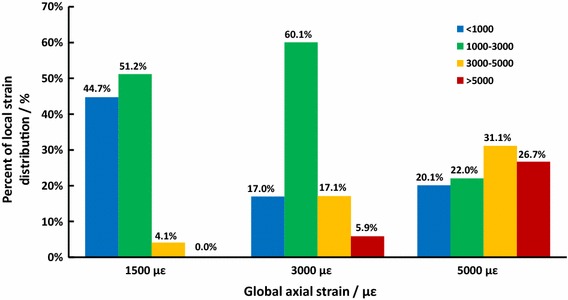
Local strain distribution in rat ulna under specific loading amplitude using FEA

Loading parameters for the athletic fatigue damage model were determined using preliminary results. The average loading frequency (f = 1.5 Hz) was calculated based on sports data and the standard statistics of male Chinese running athletes and military recruits (Table 1). The local strain distribution (Fig. 2) and fatigue behavior of the rat ulna under a specific loading intensity (ε_max_ = 3000 με) was simulated using 3D reconstruction and finite element analysis (FEA) [17]. The percent decrease in the secant modulus (E_s_) and the associated cyclic energy dissipation (H_c_) were common indices of material fatigue behavior [12, 18, 19]. In the present study, the number of fatigue loading cycles was determined by an in vitro fatigue test of rat ulnae, and we considered a 25% decrease in the E_s_ [20–22] with significant H_c_ changes as validation of the athletic fatigue damage model, which could activate bone targeted remodeling.

**Table 1:** Determination of the athletic fatigue loading frequency based on Chinese sports statistics

Objects of study	Basic information	Stride (A, cm)	Stride frequency (f, steps/min)	Loading frequency on the right leg (f_L_, Hz)
Chinese middle-distance athletes and military recruits	Gender: Male	150.2 ± 3.3	179.7 ± 3.9	1.50 ± 0.03
	Age: 18.7 ± 1.4 years			
	Height: 173.8 ± 5.1 cm			
	Velocity: 16.2 km/h

### Histology and morphometric analysis

Hematoxylin–eosin staining on non-decalcified slices of the middle sections of the ulnae were performed by XueBang Pathology Corporation in Beijing, China. A qualitative observation of ulnar micromorphology was performed on an optical microscope (Olympus, Japan), and the percentage of empty osteocyte lacunae was quantitatively analyzed and compared between different groups.

### Tests of serum protein expression and ulnar mechanical behavior

ELISA kits for TRAP-5b and BGP were used to detect bone resorption and formation, respectively. The influence of estrogen on bone remodeling [17, 23, 24] was also determined by assaying serum estradiol with an E2 ELISA Kit. The quasi-static mechanical properties of the ulnae were tested on an INSTRON 5865 (Instron Corporation, England) with the following parameters: (1) 0.5 N preload, 5 uniaxial loading cycles of displacement with a loading rate of 1%/min to a maximum of 0.4%; (2) a loading rate of 5%/min to fracture. Compressive modulus (E), strength (σ_b_) and fracture energy (H_f_) were determined. Fatigue mechanical properties, including cyclic E_s_, H_c_ and cycles to fatigue (N_f_), were evaluated based on the stress–strain (σ–ε) data recorded during fatigue loading. E_s_ was the slope of the line through the start and end points in the loading stage of the σ–ε loop. H_c_ was the area of the σ–ε loop, determined by integration, and N_f_ was the number of fatigue loading cycles at 25% E_s_ decrease, determined by interpolation.

### Statistical analysis

Data are presented as the mean ± SD and were from at least three independent experiments. The research was designed and simplified from a two-independent-variable experiment based on the theory of “mechanostat” [1]. Significant differences were evaluated with a one-way analysis of variance (ANOVA) followed by a least significant difference (LSD) *t* test. Significance was defined as *p* < 0.05.

## Results

**Fig. 3:**
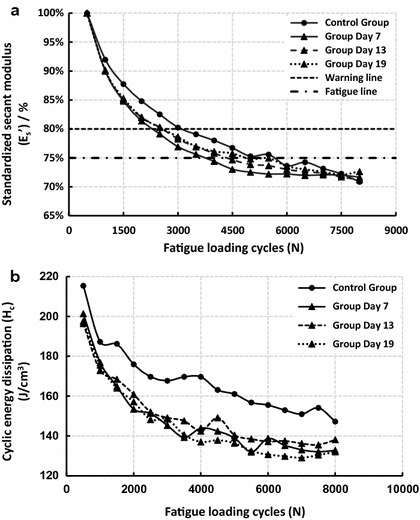
Changes of standardized secant modulus (E_s_’) (**a**) and cyclic energy dissipation (H_c_) (**b**) in different groups with fatigue loading cycles

Values of E_s_ were calculated and standardized (E_s_’) based on the 500th cycle,and curves of E_s_’–N and H_c_–N are shown in Fig. 3. The ulnae in all groups experienced fatigue successively, with E_s_’ falling to the fatigue line (25% decrease) (Fig. 3a), and there were obvious decreases in H_c_ with increasing fatigue loading cycles (Fig. 3b). Therefore, the model of athletic fatigue damage was validated.

**Fig. 4:**
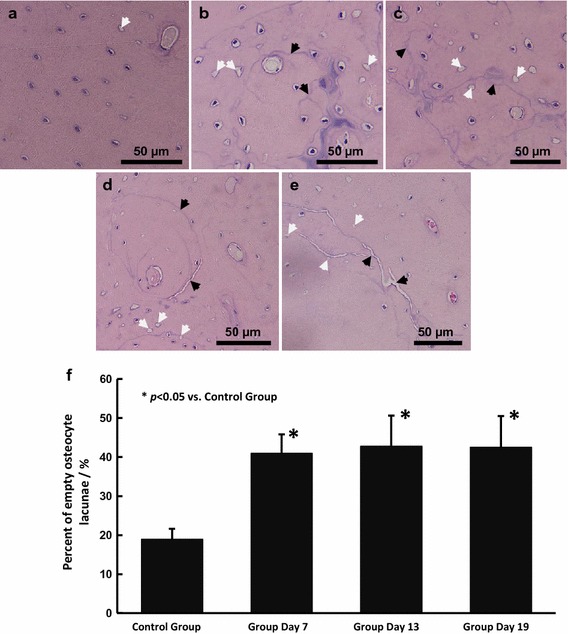
Non-decalcified slices of rat ulnae and empty osteocyte lacunae statistics. Few empty osteocyte lacunae and almost no obvious microcracks were detected in the Control Group (**a**), while generation, growth and coalescence of microcracks (*black arrows*) were observed sequentially in Group Day 7 (**b**), Group Day 13 (**c**) and Group Day 19 (**d**, **e**). Significant increases of empty osteocyte lacunae, especially around microcracks, were also detected (*white arrows*) and statistically verified (**f**)

Qualitative observation of the HE-stained, non-decalcified ulnae slices from different groups showed deterioration of the bone microstructure under athletic fatigue loading (Fig. 4). Microcrack generation (Fig. 4b), growth (Fig. 4c, d) and even coalescence (Fig. 4e) in the interstitial bone reflected an aggravation of bone fatigue damage. Significant increases in the percent of empty osteocyte lacunae were also detected (Fig. 4f) in the high intensity groups, suggesting apoptosis of osteocytes.

The concentrations of serum proteins (E2, TRAP-5b and BGP) were assayed and are listed in Table 2. No significant differences in serum E2 levels were detected; therefore, the possible influence of estrogen on bone targeted remodeling could be disregarded in our study. A significant increase in serum TRAP-5b in Day 7 suggested fatigue damage and the activation of bone targeted remodeling, and reduced serum TRAP-5b and increased serum BGP from Day 7 to Day 19 demonstrated a process of continuous bone targeted remodeling.

**Table 2:** ELISA assay of serum E2, BGP and TRAP-5b

Groups	Concentration of serum proteins (pg/ml)		
	E2	BGP	TRAP-5b
Day 7	448.58 ± 94.03	2504.26 ± 120.73*	1452.48 ± 35.38*
Day 13	512.07 ± 65.97	3669.96 ± 206.59*/^#^	1215.62 ± 52.94*/^#^
Day 19	478.53 ± 62.68	4321.60 ± 102.73*/^#^	1177.62 ± 20.71*/^#^
Control	470.18 ± 85.22	2876.21 ± 195.04	1002.46 ± 29.66

* *p* < 0.05 vs. Control group

^#^ *p* < 0.05 vs. Group Day 7

**Fig. 5:**
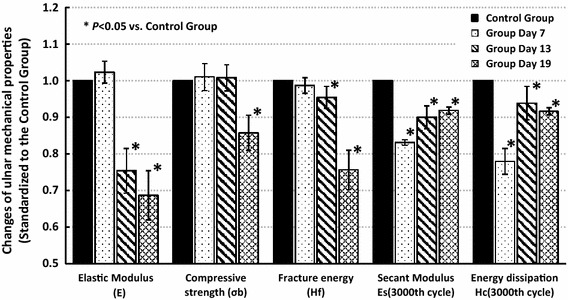
Histogram of ulnar mechanical properties in different fatigue loading groups

**Fig. 6:**
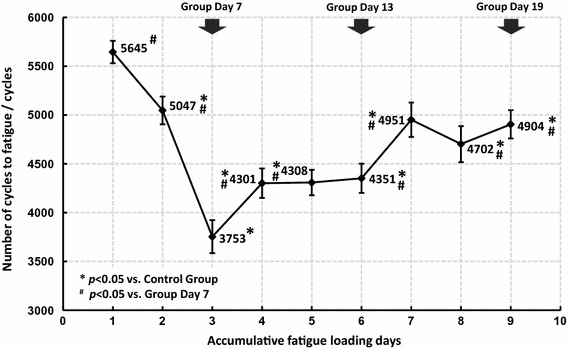
Statistical analysis of N_f_ on each loading day

Fatigue mechanical behavior of the ulnae (E_s_ and H_c_) were sensitive to fatigue loading, and significant changes were detected from Day 7, while the quasi-static mechanical properties (E, σ_b_ and H_f_) seemed to be hysteretic and did not experience significant changes until Day 13 or even Day 19 (Fig. 5). N_f_ was also statistically analyzed and the results are shown in Fig. 6. Decreases in N_f_ were detected in all fatigue loading groups, with the valley value on Day 7, which implied a rapid deterioration of the fatigue mechanical properties after fatigue loading and effective improvements by subsequent bone targeted remodeling.

**Fig. 7:**
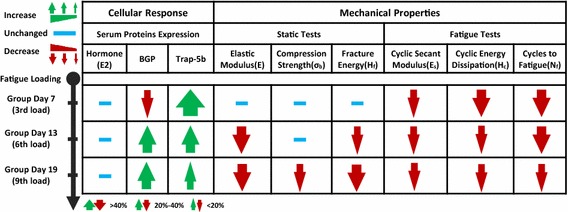
Sketch of index changes during fatigue damage induced bone targeted remodeling

Finally, we summarized the relative changes of several key indices of bone targeted remodeling at different levels (Fig. 7) and achieved a systematic understanding of the bone targeted remodeling process under athletic fatigue loading. This understanding could likely offer effective insights regarding to the training of athletes and military recruits.

## Discussion

The basic principles of bone remodeling are universal regardless of bone dimensions [1, 7], but there are huge differences in the metabolism rates of rats and humans [23]. When we determined the athletic fatigue loading parameters (loading duration, amplitude and sampling intervals), its effect on the bone targeted remodeling process had been fully considered.

We originally designed the experiment with two independent variables (periods and exercise), and simplified it (during sampling, assay and analysis) as a single variable (athletic fatigue loading duration) experiment based on Frost’s theory of the “mechanostat” [1]. Despite of the weight increase, daily activities of the 3-month-old rats in the control group could produce a physiological deformation of ulnae (with a 200–1000 με axial peak strain [25]), which would not disturb the metabolism balance between bone resorption (osteoclasts activities) and bone formation (osteoblasts activities), or lead to a detectable change of bone mechanical properties within 1 month.

Dynamic compressive loading on the long bones is common during daily activities [14, 19], and the decrease in the secant modulus is mostly a result of bone damage or microcracks [22]. The athletic fatigue loading protocol used in the present study resulted in significant decreases in bone fatigue mechanical properties (Figs. 3, 5 and 6); the generation of microcracks in the interstitial bone (Fig. 4b); and an imbalance between bone resorption and bone formation (Table 2) in the early stage (Group Day 7). Our hypothesis on bone fatigue damage and the associated targeted remodeling was therefore confirmed.

An osteon in the cortical bone acts not only as a stress concentrator that accounts for microcrack generation [25–27] but also as a guard against microcrack growth to maintain the load-bearing capacity of the bone during daily activity [28, 29]. During continuous vigorous activity, however, microcracks will grow and even develop to the macro scale, leading to a stress fracture [9, 21, 30]. Our study supported these results. From Day 7 to Day 13, small microcracks were observed (Fig. 4b, c), and fatigue mechanical properties also improved (Figs. 5, 6). At Day 19, larger microcracks grew and coalesced (Fig. 4d, e), and significant decreases in quasi-static mechanical properties were detected (Fig. 5), which affected the load-bearing capacity and increased the risk of stress fractures.

The integrity of osteocytes is vital for the structural and functional stability of bone [31]. There seems to be a threshold of microdamage or local strain [16] over which fatigue loading or the necessity of bone targeted remodeling could lead to the apoptosis of osteocytes [23, 24, 32]. In the current study, we confirmed the stable apoptosis of osteocytes in the high-intensity groups during fatigue loading through the significant increase in the percentage of empty osteocyte lacunae (Fig. 4f) along the microcracks near osteons (Fig. 4b–d) when compared with the Control group. However, there might be different mechanisms of osteocyte apoptosis in different groups. The apoptosis observed at Day 7 and Day 13 might result from the necessity of bone targeted remodeling at the early stage because small microcracks and active bone resorption were detected. By contrast, at Day 19, larger linear microcracks were observed despite hyperactive bone formation; therefore, it was likely the severe fatigue damage in the interstitial bone that sequentially prompted osteocyte apoptosis.

Finally, the responses of the bone at different levels under various mechanical conditions should compose a unified model. We achieved a systematic understanding of the process of bone targeted remodeling under athletic fatigue loading, as illustrated in Fig. 7. Strenuous activity in the early stage (3 days in rats or 1 month in humans [23]) would cause rapid microdamage, decreases in fatigue mechanical properties and obvious bone resorption in the long bones. With increasing time, the risks of stress fracture would increase with the deterioration of the quasi-static mechanical properties of the bone despite a continuous bone targeted remodeling process, suggesting a limitation or a maximum ability to repair excessively damaged bone. For this reason, excessive early-stage training or long-term intensive training without appropriate rests should be avoided to prevent the risk of accumulative fatigue damage or even stress fractures.

## Conclusion

An athletic fatigue damage model of rat ulna was successfully established. Fatigue damage was aggravated in the loading process, with osteocyte apoptosis, microcrack accumulation, and a decrease in mechanical properties. Bone targeted remodeling was activated after athletic fatigue loading and progressively leaned towards bone formation and away from bone resorption to repair fatigue damage, though to some extent.

## Abbreviations

OBs: osteoblasts
OCs: osteoclasts
E2: estradiol
BGP: bone gamma-carboxyglutamic-acid-containing protein
TRAP-5b: tartrate-resistant acid phosphatase 5b
FEA: finite element analysis
